# Exploring the Relationship Between Subjective Age and Worry for Older Adults in Times of a Pandemic

**DOI:** 10.1093/geroni/igab046.2271

**Published:** 2021-12-17

**Authors:** Maiken Tingvold, Anna Kornadt, Isabelle Albert, Elke Murdock, Martine Hoffmann, Josepha Nell

**Affiliations:** 1 University of Luxembourg, University of Luxembourg, Grevenmacher, Luxembourg; 2 University of Luxembourg, Esch-sur-Alzette, Luxembourg; 3 University of Luxembourg, Esch-sur-Alzette, Diekirch, Luxembourg; 4 RBS, Itzig, Diekirch, Luxembourg; 5 University of Luxembourg, Esch-sur-Alzette, Grevenmacher, Luxembourg

## Abstract

Given the role of age as a risk factor in the covid pandemic, we examined the longitudinal cross-lagged relationship between subjective age and Covid-related worry, and possible moderators of this relationship. Data were obtained at two-time points (June and October 2020) by a phone/online survey, from N = 611 older participants (Mage = 69.92 years). Participants felt on average 10 and 8.5 years younger than their chronological ages at the two-time points, respectively. Younger subjective age at T1 increased the level of worry at T2 irrespective of age, perceived control and subjective health. Higher worry increased subjective age at T2, but only for those with worse subjective health. Our results show that subjective age and Covid-related worry interact over time. This relation needs to be explored further in order to understand the relationship between subjective age and well-being especially, but not only in the pandemic context.

